# TLR4 mediates inflammation and hepatic fibrosis induced by chronic intermittent hypoxia in rats

**DOI:** 10.3892/mmr.2021.12144

**Published:** 2021-05-10

**Authors:** Zhi-Peng Lin, Hui-Li Lin, Xue-Ping Yu, Yi-Juan Zheng, Si-Yu Cheng

Mol Med Rep 22: 651-660, 2020; DOI: 10.3892/mmr.2020.11134

Following the publication of the above article, the authors have realized that the first grant number featured in the Funding section of the Declarations on p. 658 appeared incorrectly: The text here should have been written as ‘grant nos. **2018J01199**, 2018Y0032 and 2016J01441′ instead of ‘grant nos. 2018J0105, 2018Y0032 and 2016J01441′.

The authors regret their oversight in providing this incorrect information in the Funding section of their paper. They thank the Editor of *Molecular Medicine Reports* for allowing them the opportunity to publish this corrigendum, and apologize to the readership of the Journal and to the funding body in question for any inconvenience caused.

